# Nano-photoluminescence of natural anyon molecules and topological quantum computation

**DOI:** 10.1038/s41598-021-00859-6

**Published:** 2021-11-02

**Authors:** Alexander M. Mintairov, Dmitrii V. Lebedev, Alexei S. Vlasov, Alexei O. Orlov, Gregory L. Snider, Steven A. Blundell

**Affiliations:** 1 Ioffe Institute, Saint Petersburg, 194021 Russia; 2 Electrical Engineering, University of Notre Dame, Notre Dame, IN 46556 USA; 3 University Grenoble Alpes, CEA, CNRS, IRIG, SyMMES, 38000 Grenoble, France

**Keywords:** Quantum dots, Quantum Hall

## Abstract

The proposal of fault-tolerant quantum computations, which promise to dramatically improve the operation of quantum computers and to accelerate the development of the compact hardware for them, is based on topological quantum field theories, which rely on the existence in Nature of physical systems described by a Lagrangian containing a non-Abelian (NA) topological term. These are solid-state systems having two-dimensional electrons, which are coupled to magnetic-flux-quanta vortexes, forming complex particles, known as anyons. Topological quantum computing (TQC) operations thus represent a physical realization of the mathematical operations involving NA representations of a braid group *B*_*n*_, generated by a set of *n* localized anyons, which can be braided and fused using a “tweezer” and controlled by a detector. For most of the potential TQC material systems known so far, which are 2D-electron–gas semiconductor structure at high magnetic field and a variety of hybrid superconductor/topological-material heterostructures, the realization of anyon localization versus tweezing and detecting meets serious obstacles, chief among which are the necessity of using current control, i.e., mobile particles, of the TQC operations and high density electron puddles (containing thousands of electrons) to generate a single vortex. Here we demonstrate a novel system, in which these obstacles can be overcome, and in which vortexes are generated by a single electron. This is a ~ 150 nm size many electron InP/GaInP_2_ self-organized quantum dot, in which molecules, consisting of a few localized anyons, are naturally formed and exist at zero external magnetic field. We used high-spatial-resolution scanning magneto-photoluminescence spectroscopy measurements of a set of the dots having five and six electrons, together with many-body quantum mechanical calculations to demonstrate spontaneous formation of the anyon magneto-electron particles (*e*^*ν*^) having fractional charge *ν* = *n*/*k,* where *n* = 1–4 and *k* = 3–15 are the number of electrons and vortexes, respectively, arranged in molecular structures having a built-in (internal) magnetic field of 6–12 T. Using direct imaging of the molecular configurations we observed fusion and braiding of *e*^*ν*^*-*anyons under photo-excitation and revealed the possibility of using charge sensing for their control. Our investigations show that InP/GaInP_2_ anyon-molecule QDs, which have intrinsic transformations of localized *e*^*ν*^*-*anyons compatible with TQC operations and capable of being probed by charge sensing, are very promising for the realization of TQC.

## Introduction

Methods of topological quantum computation (TQC) are based on topological quantum field theories, which show that two-dimensional (2D) electron systems having magnetic-flux-vortexes can be modeled by a Hamiltonian whose eigenstates correspond to the states of quantum error correcting code^1^. This also means that they are described by an effective Lagrangian containing a non-Abelian (NA) topological term^2^. According to the theory, the quantum computation process in these systems will be protected from environmental distortion at the physical level and will be intrinsically fault-tolerant. Topological quantum-gate (TQG) processing represents a physical realization of mathematical operations involving NA representations of a braid group *B*_n_ generated by a set of *n* localized particles (anyons) having zero-energy excitations, which are Majorana zero modes (MZMs), each representing a qubit state. A MZM qubit involves magnetic-flux-vortexes having opposite directions, representing a particle-antiparticle pair. The operation of the gate includes moving particles around each other (braiding) and pairing (fusion) together with the generation of the particles and the control of the resulting state^1,3^. Up to now, all efforts to realize TQC have been focused on finding an appropriate system having anyons with MZMs^4–9^. Although it is not clear how the gate operation will be physically realized using the specific systems investigated^4,10^, it should generally involve some local potential perturbations for particle trapping and moving, i.e., “tweezing” ^11–13^. This tweezing procedure, changing the TQG qubit state, is the equivalent of the resonant electro-magnetic pulses that induce Rabi oscillations of a two-level qubit state in the conventional schemes of quantum computing (QC)^14^. However, while the MZM-qubit and tweezing are supposed to be topologically protected, two-level qubits and Rabi oscillations are not, and they require adding redundant qubits to permit error-correcting code processing. This processing should provide an extremely low error-probability (fidelity) threshold to make QC operations fault-tolerant^15^. Such a threshold has been demonstrated only for a single qubit in the major QC platforms developed so far, which are Josephson tunnel junctions^16^ and electron-spin qubits in the different solid state environment or ion traps configuration^17–21^, but for two or more qubits the fidelity is poorer^22^. Thus, the realization of TQC is of high demand.

TQC relies on the existence of anions, which are composite particles consisting of electrons and a few magnetic-flux-quanta vortexes^23^. The anyon has fractional charge and its wave-function can have an arbitrary phase after interchange. Multi-dimensional NA representations of *B*_*n*_ are formed by a set of *n* “coupled” anyons having a strongly degenerate ground state^24^. The wavefunction in this case is a vector depending on the position and the quantum number of each particle; particle exchange then gives a matrix, i.e. a NA transformation of this vector, which is topologically protected. Theoretically, NA anyons can be formed with half-vortexes in *p*-wave superconductors, which have MZM in the core ^25–27^.

The richest and most investigated anyon system is a 2D-electron semiconductor heterostructure in a perpendicular magnetic field in which anyon states are formed at fractional Landau level (LL) fillings and provide a dissipationless skipping-type edge current in conjunction with a transverse conductance plateau known as the fractional quantum Hall effect (FQHE)^28^. More than 50 FQHE anyon states were observed in the lowest Landau level (LLL)^29^ and about 14 states in the second Landau level (SLL)^30^. NA anyons have been suggested and probed for the state *ν* = 5/2 having half-filled *p*-wave state at SLL^31–33^. For the hypothetical SLL NA state*ν* = 12/5 the braids that yield a universal set of quantum gates were found^3^. FQHE anyons are probed by the edge currents involving hundreds of mobile particles, which make difficult direct TQC.

Another type of structures suggested for TQC are hybrid heterostructures, in which half-vortexes are expected to form using the proximity effect between a conventional *s*-wave superconductor and various topological structures/materials, such as semiconductor nanowires having strong a spin–orbit coupling^5^, chains of ferromagnetic atoms^6^, topological insulators^7^, anomalous Hall insulator–superconductor^8^, and an insulating 2D quantum magnet^9^. The key difference between these systems and the FQHE one is that a single vortex is formed by up to a few thousands of electrons forming normal core at the center of a superconducting current loop^7^, and because of this “massive” structure, tweezing seems to be hard to realize in these systems. The last two systems use edge currents and have the same difficulties as FQHE system.

Currently FQHE anyons are considered theoretically to be quasi-particle excitations of the host ground-state liquid ^34^ and the quasi-particle picture is the basis for topological quantum field theories describing specific NA states and their use for building qubit and gate operations^3,8^. We have shown recently, however, that the FQHE anyon can exist as a single localized particles, which do not involve many-body interactions for their formation nor require quasi-particle concept for their description. Such fractionally charge particles were proposed to explain magneto-photoluminescence (magneto-PL) measurements of quasi-2D InP/GaInP_2_ single-electron islands (quantum dots) having Wigner–Seitz radius *r*_s_ ~ 4 ^35^. In this particle, which we called a magneto-electron (*e*^*ν*^), a corresponding number of magnetic-quantum-flux vortexes (*k* = 1/*ν*) are self-generated.

Here we report the observation of molecular structures of *e*^*ν*^s using magneto-PL measurements of QDs having about six-electrons and *r*_s_ ~ 2 and show that these *e*^*ν*^-anyon molecules (*e*^*ν*^-AM) represent a novel system, which can be used for the realization of TQGs. The measurements include the imaging of the emission area of individual PL lines, together with quantum mechanical calculations and analysis of their electronic structure in a magnetic field. Using these we demonstrate the self-formation of *e*^*ν*^-AMs and report observation of the *e*^*ν*^-AMs having *ν* ~ 3/5–1/4, corresponding to a built-in magnetic field 6–12 T. We also observe a transformation of the *e*^*ν*^ arrangement in a 3 *e*^2/7^-AM under photo-excitation demonstrating fusion and braiding of the anyons. The observed transformation reveals a significant redistribution of the fractional charge within the dot, which suggests the use of single electron transistor charge sensing to control the TQC operations.

## Experimental details and data

### InP/GaInP_2_ quantum dots

We have studied nine single self-assembled InP/GaInP_2_ QDs^36^ having a lateral size of *D* ~ 120 nm and five-to-six electrons using near-field scanning optical microscope (NSOM) at temperature 10 K, external magnetic field range *B*_e_ = 0–10 T and spatial resolution up to 25 nm (see “Methods”).

The dots were selected using NSOM measurements of a set of about 200 dots in variety of InP/GaInP_2_ QDs structures in previous studies^36–38^. Specifically, nine dots investigated were obtained in the sample X3141 (see Ref.^36^) and all of them have “anomalous” PL spectral features revealed in Refs.^36,37,39^ in comparison with the “normal” dots typically having more electrons and smaller size (see below). Two of such dots, also discussed here, were measured in the same sample.

All dots (except one normal) are denoted by D*kx*, where the letter D indicates the initial identification of the shell filling (see the dot D5 in Ref.^36^, which is labeled D4e dot here), *k* enumerates the dots and *x* = a, b, c … indicates the NSOM run. One normal dot is denoted F1e. The dots are located within an area ~ 2 × 2 µm^2^ for the same run/scan (see below) and within an area ~ 2 × 2 mm^2^ for different runs.

For anomalous dots the parameters measured include the AM type, the main peak energy *E*_0_, the *s-p* splitting Δ*E*_sp_, the size *D*, the Wigner–Seitz radius *r*_s_, the built-in magnetic field *B*_bi_, *ν* and the *e*^*ν*^ configuration. The AM type, *E*_0_ and Δ*E*_sp_, were measured from intensity distribution, position and energy splitting of the PL spectral components. The *D* values for most of the dots were measured/estimated directly from scanning experiments and used for calculation of *r*_s_ = *DN*^-0.5^/(2*a*_B_^*^), where *a*_B_^*^ ~ 8 nm^35^. *B*_bi_ and *ν* was estimated from a complex analysis of the whole data set including the dependence of the PL spectra on *B*_e_, the NSOM maps and theoretical calculations/analysis (see below). The *e*^*ν*^ configuration was suggested from analysis of the whole data set. These parameters apply to the photo-excited state (PS), which for all dots (except D1e) have N* = *N* + 1 = 6 electrons, where *N* is number of electrons in initial (IS) state. For D1e N* = 7.

Table 1 summarizes the dot parameters measured. The parameters, important for further discussion, are *r*_s_, which changes from 2.2 to 2.6 making up 20% variations and *B*_bi_, which changes from 6 to 12 T making up two times (200%) variations.

**Table 1:** Parameters of AM states of InP/GaInP_2_ QDs measured using NSOM.

#	Name	AM type	*E*_0_ (eV)	*ΔE*_*sp*_ (meV)	*D* (nm)	*r*_s_	$B_{{{\text{bi}}}} \;({\text{T}})$	*ν*	*e*^*ν*^ molecular state PS(IS)
1	D2b	AM_m_	1.7063	1.9	~ 110	2.3	9	1/3	${6e}^{1/3}$
2	D4b	AM_m_	1.6870	1.7	~ 120	2.35	11	2/7	${3e}^{2/7}$
3	D5b	AM_6,0_	1.7143	1.7	~ 130	2.4	11	3/11	${2e}^{3/11}$
4	D1d	AM_5,1_	1.7075	2.0	~ 110	2.2	6	3/5	${2e}^{3/5}$
5	D5d	AM_5,1_	1.7043	2.0	~ 110	2.2	6	3/5	${6e}^{1/3}$
6	D1e	AM_6,0_	1.7686	1.7	150/70^a^	2.4	11	4/15	${3e}^{1/3}+{e}^{4/15}({3e}^{2/7})$
7	D2e	AM_m_	1.7293	1.4	~ 140	2.6	12	3/13	${2e}^{3/14}$
8	D4e	AM_6,0_	1.7311	1.6	120/70	2.5	12	1/4	${6e}^{1/4}({5e}^{1/4})$
9	D5e	AM_m_	1.7913	1.6	~ 135	2.5	12	1/4	${6e}^{1/4}$

### Theoretical description

The analysis of the experimental data was done using a phenomenological description within a framework of a general theory based on exact quantum mechanical calculations involving Fock–Darwin (FD), Hartree–Fock (HF) and configuration interaction (CI) approaches, developed for a few 2D electrons confined in circular potential in a perpendicular magnetic field about 20 years ago ^40–42^.

The many-body HF and CI methods were used to calculate dependence of the energy structure together with the shell energy and electron distribution on magnetic field for specific QD studied taking into account their shape, and, thus, a circular symmetry breaking effects (see “Methods”). These were used for analysis and comparison with corresponding experimental data.

Single-particle FD spectrum for circular symmetric dots^43^ was used in the form *E*_*k,l*_(*B*) = *E*_*k,l*_(*B*_e_ + *B*_bi_), where *E*_*k,l*_(*B*) = *E*_*k,l*_^FD^(*Ω*) = *ħΩ*(*k* + 1) − 1/2*ħω*_c_*l*, and *Ω*^2^ = *ω*_0_^2^ + *ω*_c_^2^/4, where *ω*_0_ and *ω*_c_ = e*B/m* are the quantum confinement and cyclotron frequencies, respectively, and *k* and *l* are the principal (*k* = 0, 1, ..) and azimuthal (*l* = -*k*, -*k* + 2, …, *k*-2) quantum numbers. We used an approximate expression for *E*_*0,0*_(*B*_0_) $\approx$ N*V_c_ derived in Ref.^38^ for *N* = 1, where *ν*_c=_0.07*ω*_c_(m*Ω)^−0.5^ is a matrix element of the Coulomb interaction.

The FD spectrum was used to fit the experimental shifts of PL lines versus external magnetic field *B*_e_ to estimate the electron charge e* and *B*_bi_.

### Photo-luminescence spectra

#### Anomalous spectral features and intensity distributions

**Figure 1:**
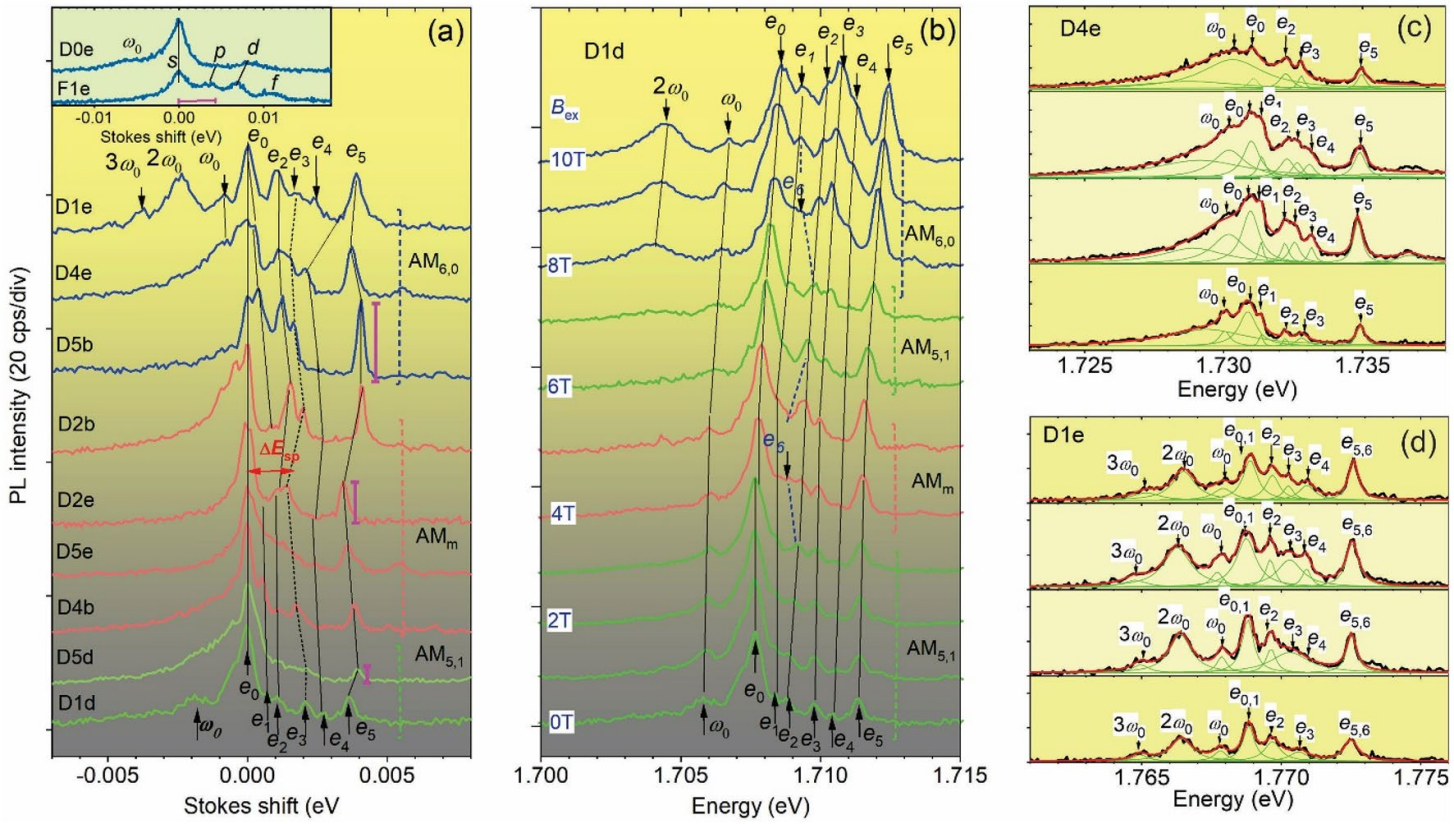
PL spectra of D*kx* anomalous InP/GaInP_2_ QDs at zero external magnetic field *B*_e_. Solid lines connect the corresponding PL peaks, dashed ones connect peak *e*_3_. The upper insert shows PL spectra of two normal dots D0e (*N* ~ 8, Δ*E*_sp_ ~ 4.5 meV) and F1e (*N* ~ 12, Δ*E*_sp_ ~ 3.5 meV) and the horizontal bar indicates the spectral range of anomalous dots (**a)**. PL spectra of the QD D1d in external magnetic fields *B*_e_ = 0, 1, 2 … and 10 T **(b)**. Spatially resolved NSOM spectra (*B*_e_ = 0 T) of D4e **(c)** and D1e **(d)** QDs, respectively, taken at tip positions separated by 50 nm.

The spectra of anomalous dots presented in Fig. 1a have a set of sharp peaks, which are a main zero-energy *e*_0_-peak and about five *e*_1_- *e*_5_ peaks, related to anti-Stokes components (ASCs) having a splitting of ~ 0.5 meV. This is significantly different from the spectra of normal dots presented in the insert (see figure caption for their parameters), which reveal two or three ASC peaks, a few times larger splitting (3–5 meV), and order-of-magnitude larger peak broadening. While, in the normal dots, the zero-energy and ASCs peaks are related to the occupied degenerate *s*- *p*-, *d*-, … electron (*e*) shells (including a photo-excited *e*), in the anomalous ones they are related to single spin-polarized *e*s and manifest *B*_bi_, as we will show below. The energy splitting between the *e*_0_ and the *e*_3_ peaks Δ*E*_sp_, shown by a horizontal arrow, is related to the *s*-*p*-shell splitting, which, according to HF calculations, determines a quantum confinement energy *ħω*_0_^*^ = 0.7Δ*E*_sp_. Besides the ASCs, Stokes components (SCs) denoted as *nw*_0_ (*n* = 1–3, *ħω*_0_ $\approx$ Δ*E*_sp_) are observed for some dots (D1d, D2b, D4e and D1e) and they are related to a center-of-mass vibration.

While the anomalous spectral features are nearly the same for all dots, the intensity distribution of ASCs shows significant variations. These variations reveal three types denoted by AM_5,1_, AM_m_ and AM_6,0_. For the AM_5,1_ type (see two lowest spectra in Fig. 1a) the ASCs are an order of magnitude weaker than *e*_0_. For the AM_m_ type (four middle spectra) the intensity of the ASCs increases a few times reaching a value of up to half of *e*_0_. For the AM_6,0_ type (see three upper spectra) the intensity of the ASC peaks increases further and becomes nearly the same as the intensity of the peak *e*_0_.

### Magnetic field dependence

In the magnetic fields *B*_e_ = 0–10 T (see Fig. 1b) the AM_5,1_-type D1d dot reveals a very weak diamagnetic shifts of PL spectral lines (about 0.5 meV for 10 T) and a strong change in relative intensity versus *B*_e_ resulting in the appearance of the type AM_m_ at *B*_e_ = 4–5 T and type AM_6,0_ at 8–10 T. The emergence of these two types is accompanied by the appearance of an additional peak *e*_6_ between peaks *e*_1_ and *e*_2_. For the AM_6,0_ type a SC at 2*ω*_0_ appears, similar to the dot D1e (see Fig. 1a).

For normal dots the shifts (not shown here) are an order of magnitude stronger (up to 1 meV/T) having anti-crossings and paramagnetic regions at fields < 2 T , while the intensity distributions do not show significant changes, as will be discussed elsewhere.

### Spatially-resolve spectra and imaging

**Figure 2:**
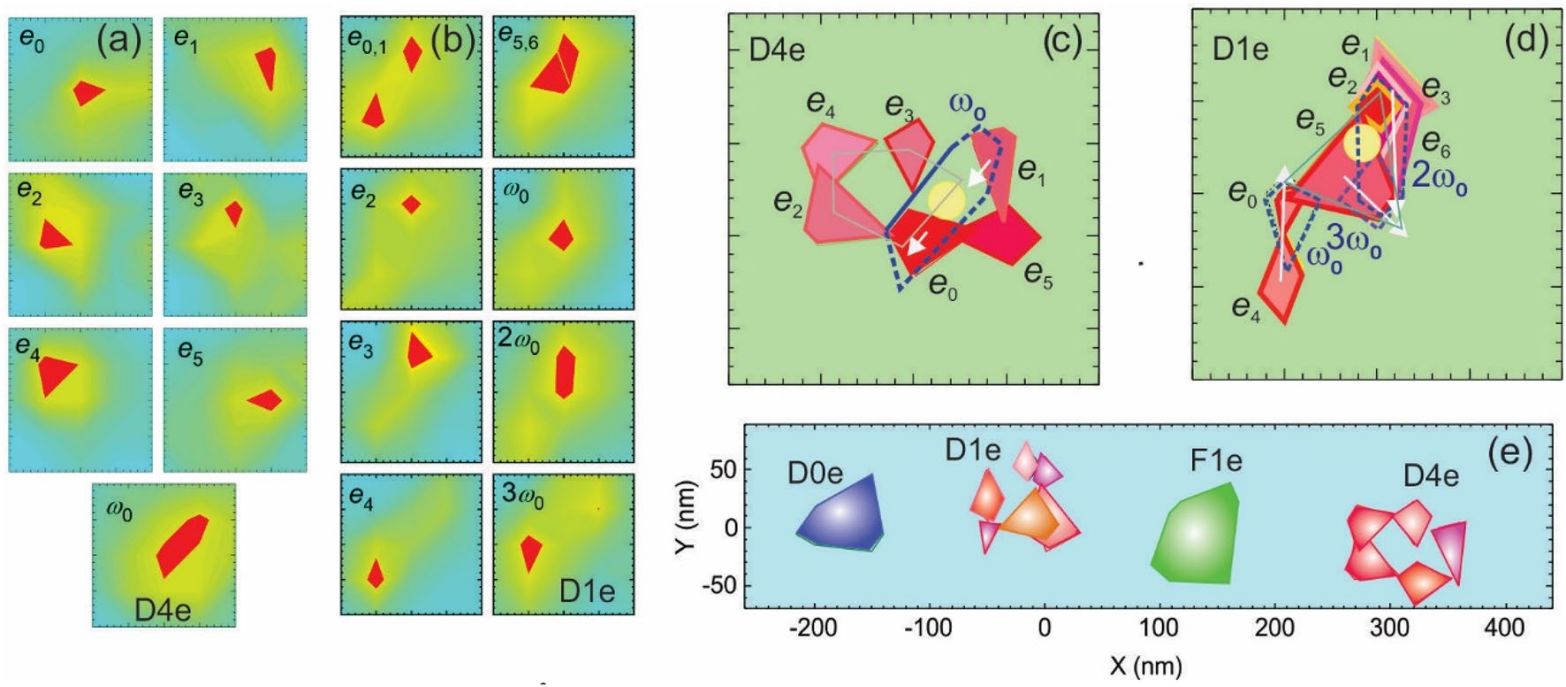
NSOM intensity images (size 200 × 200 nm^2^) of the individual spectral lines of D4e **(a)** and D1e **(b)** QDs, respectively. Combined EAs of a and b, respectively (**c, d**). Solid shapes and dashed contours are ASCs and SCs, respectively. Arrows show the shift of the ASC EAs in the IS and a circle marks the expected location of a photo-excited hole. Dark–light color code of ASCs outlines line intensity. Combined maps of D0e, D1e, F1e and D4e in IS (**e)**.

The dots reveal anomalous size of the emission area (EA) of the single-*e* PL peaks, which is seen from spatially resolved data for dots D1e and D4e (see Fig. 1c,d and Fig. 2a–e). A variation of the intensity of *e*_0_-*e*_5_ peaks down to ~ 0.4% per nm is seen in spectra in Fig. 1c,d. This corresponds to an EA size *d*_EA_ down to ~ 25 nm as it is seen in the PL intensity maps in Fig. 2a,b, giving *d*_EA_ = 40 ± 20 nm.

The maps also show the location of the individual *e*s, and the combined maps presented in Fig. 2c,d display molecular structures. The molecular structure is absent in the normal D0e and F1e dots, the EA of which corresponds to the dot size of ~ 90 and 110 nm, respectively. This is seen in the combined map of the area containing these four dots and presented in Fig. 2e. In the map the images of IS are presented for D1e and D4e dots. The map shows that these are neighboring dots arranged in a liner chain with separation ~ 200 nm.

The molecular structure of the QD D4e in PS in Fig. 2c is a compressed hexagon having a size ~ 120 × 70 nm and a bond length ~ 40 nm. The IS structure (see Fig. 2e) is a pentagon, in which *e*_0_ and *e*_1_ electrons are shifted by 10–20 nm along the (*x,y*) diagonal, as follows from *ω*_0_ EAs shape.

A complicated quasi-1D molecular structure oriented along the (*x,y*)-diagonal and having a size ~ 150 × 70 nm^2^ is observed for D1e (see Fig. 2d). In this structure *e*_1_, *e*_2_, *e*_3_ and *e*_6_ EAs overlap and *e*_0,1_ and *e*_5,6_ EAs have two locations, revealing degenerate 2*e* states, which gives a 7*e* PS of this dot. The overlapped peaks are located in the upper right corner of the map; the rest three peaks are located along the (*x,y*)-diagonal below at the distance ~ 70 nm and separated from each other by 40 nm.

The SCs maps correspond to a ~ 60 nm vertical up shift of *e*_4_ (*ω*_0_ map), a ~ 60 nm down shift *e*_3_ (2*ω*_0_ map) and a ~ 40 nm down shift of *e*_5_ along the (*x,-y*)-diagonal (2*ω*_0_ map). This results in *e*_0_-*e*_4_ and *e*_3_-*e*_5_ pairing and in the IS the molecular structure has nearly equilateral triangle arrangement of paired *e*s at the vertexes, having sides ~ 80 nm and 90 nm and a bond length ~ 60 nm.

In the combined maps in Fig. 2c,d a dark–light color code of EAs corresponds to large-small separation of *e*s from the photo-excited hole, which allows determine its location as shown in the maps. Thus, specific spectral shape observed is related to a specific distribution of such separations (see below).

### Analysis of the data

#### Classification of energies and states in magnetic field

**Figure 3:**
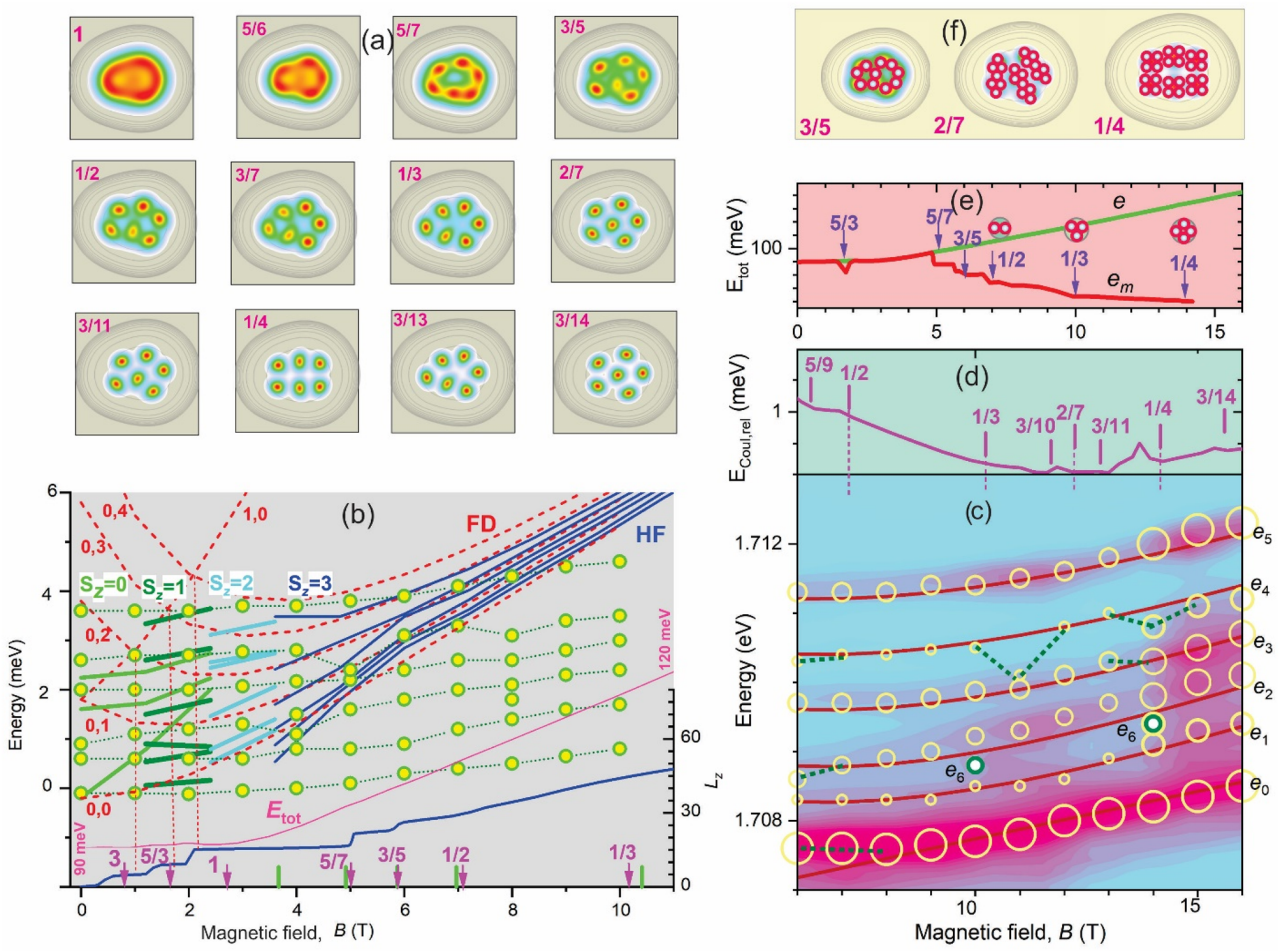
Calculated electron density distributions given by CI of the QD D1d for filling factors (numbers in the plots) *ν* = 1, 5/6, 5/7, 3/5, ½,3/7, 1/3, 2/7, 3/11, ¼, 3/13 and 3/14. Contours indicate the deformed confinement potential (**a)**. Calculated total energy (thin line) ranging from 90 meV at B = 0 T to 120 meV at B = 12 T, total angular momentum *L*_*z*_ (thick line, axis on right side) and shifts given by FD (dashed lines) and by HF *S*_z_ = 0–3 (solid lines) versus a magnetic field range 0–10 T. Vertical dashed lines mark the crossing of the 1,0 and the 0,*l* FD levels. Solid circles are the experimental shifts of ASCs. The vertical arrows and bars on *B*-axis are *B*_*ν*_ “spectrum” of non-circular and circular dot (see text), respectively (**b)**. PL spectra (contour plot) and peak shifts (open circles) versus total field *B* and FD fit (curves) (**c)**. *E*_Coul,rel_(*B*) **(d)**. *e* versus *e*^*ν*^ *E*_tot_(*B*) and vortexes occupying single-*e* area (circles) for *ν* = 1/2, 1/3 and ¼ (**e)**. Schematic *e*^*ν*^ molecular arrangement for the QDs D4d, D1e and De4 having *ν* = 3/5, 2/7 and ¼, respectively **(f)**.

A general theory of circular dots having *N* electrons in a magnetic field distinguishes four contributions to the total energy *E*_tot_, first discussed for 2*e* in Ref.^44^, which are the kinetic energies of localization induced by the confinement potential *E*_conf_(*N*) and by the magnetic field, i.e., cyclotron motion, *E*_cycl_(*N*,*B*), respectively, and the Coulomb energies of electrons center-of-mass *E*_Coul,c.m_(*N*) and relative *E*_Coul,rel_(*N*,*B*) motion, respectively. The *B-*independent parts are *E*_conf_(*N*) = *K*(*N*)*ħω*_0_^*^ and *E*_Coul,c.m_(*N*) *r*_s_*E*_conf_(*N*). The *B-*dependent cyclotron term *E*_cycl_(*N*,*B*) = *ħω*_c_N*/2 is dominant for large fields when *ω*_c_ > *ω*_0_. The *B*-dependent term *E*_Coul,rel_(*N*,*B*) is non-trivial and, in spite of its extremely small value ~ 0.01*E*_Coul,c.m_ (see Fig. 3d below), provides ground-state transitions having discrete total angular momentum values *L*_*z*_(*B*) = ${\sum }_{i}^{N}{l}_{zi}$, where *l*_zi_ is the angular momentum of single-*e*s, known as magic numbers (*L*_*z*_^MN^). These appear because of the necessity of matching the spatial structure of the *e* wave-functions and the spatial symmetry of the *e* arrangement^45^. The *e* state of the specific *L*_*z*_^MN^ is related to a filling factor *ν* = *L*_0_/*L*_z_^MN^, where *L*_0_ = *N*(*N* − 1)/2, linking it to FQHE^46^. The set of values *B*(*L*_z_^MN^) = *B*_*ν*_ represent a “spectrum”, which for specific *N* value, is scaled as ~ 1/*r*_s_.

For a circular symmetry analog of the dot D1d, for which *E*_tot_(0) = 90 meV, *E*_cycl_(*B*) = 4.2/*B* (meV/T) and Δ*L*_*z*_ = 5 or 6 ^46^, respectively, the molecular states are spin-polarized, *ν* < 1-states and are developed at *B* > 4 T, after maximum density droplet formation ^47^. In the range *B* = 5–14 T, embracing *ν* *=* 1/2–3/10, the dominant configuration is (5,1)^46^.

### Molecular configurations and PL spectra intensity distributions

In the QD D1d the mixing of the configurations takes place, due to non-circular shape, as is seen in electron density distributions, calculated using CI, in Fig. 3a. Nine density distributions shown for *ν* from 1 to 3/14 occupying *B* range 2.7–16 T reveal the onset of molecular structure formation near *ν* = 5/7 (*B* ~ 5 T) similar to the circular symmetry. A “pure” (6,0) and (5,1) configurations appear for *ν* = 5/7 and ¼ and for *ν* = 2/7 and 3/14, respectively. For other values of *ν* two mixed configurations are appeared. In the one (see *ν* *=* 1/2) a central *e* maximum is shifted ~ 10–20 nm down from the center, and in the other (see *ν* *=* 1/3) a pentagon with adjacent single-*e* maxima at the left is formed. Note, that at *ν* = 1/4 the calculated configuration is a compressed hexagon, exactly the same as observed experimentally in D1e, which implies *B*_bi_ ~ 12 T.

Molecular configurations in Fig. 3a can be assigned to specific AM types spectra observed in Fig. 1a,b. They, together with the maps in Fig. 2c,d, show that the appearance of different AM types spectra reflects a competition between (6,0) and (5,1) isomers for a non-circular dot shape and different *B*_bi_ or *B*. In these the former has nearly equal AM_6,0_ type ASC intensity, due to the nearly equal separation of electrons from the hole, while the latter has one dominant peak AM_5,1_ type owing to its location closer to the hole and the AM_m_-type has intermediate, mixed configurations and intensity distributions.

### Magneto-electrons and spontaneous anyon molecule formation

The analysis of the calculated *B*-dispersion of HF and FD shell energies, *L*_*z*_, *E*_tot_ and their comparison with experimental data of the dot D1d shown in Fig. 3b together with the FD fit to these data in Fig. 3c and *E*_Coul,rel_ and *E*_tot_, shown in Figs. 3d,e, respectively, reveal the formation of the fractionally charged *e*^*ν*^s.

The *E*_tot_ curve in Fig. 3b shows a nearly dispersionless *E*_conf_(*N*) + *E*_Coul,c.m_(*N*) contribution for *B* < 3 T, a linear increase from *E*_cycl_(*N*,*B*) and *B* > 4 T, and weak oscillations from *E*_Coul,rel_(*B*) at *B*_*ν*_ (see also plot of *E*_Coul,rel_(*B*) in Fig. 3d) over the entire *B*-range. The *L*_*z*_^MN^ states of the dot D1d are clearly visible on *L*_*z*_*(B*) curve as plateaus and weak kinks at *L*_*z*_ = 5, 9, 15, 21, 25 30, 35 and 45. The corresponding *B*_*ν*_-spectrum has a *ν* values set from 3 to 1/3 and is very close to that of circular dot^46^. The plateaus are also seen in the region *ν* > 1corresponding to the integer (*ν* *=* 3) and SLL fractional (*ν* *=* 5/3) QHE states, demonstrating its deep connection to the localized *e*s states in QDs.

This connection is also revealed for FD states, which appears as a matching of the crossings between the (1,0) and (0,*l*) FD levels to *L*_*z*_^MN^ transitions at *ν* = 3 to 5/3 and to 1, corresponding to a total spin transitions from *S*_z_ = 0 to 1 to 2. The HF energies, accounting for spin and *p*_*x*_-*p*_*y*_ circular symmetry distortion splittings, are shown in the figure in corresponding regions and approximately match the FD energies, neglecting the splitting. Both HF and FD energies at large fields come out to the LLL line *ħω*_c_/2, with a nearly order-of-magnitude reduction of inter level splitting, which evolves to zero in the limit *B *$\to \infty$.

From Fig. 3b we can see, that the critical discrepancy between the calculated FD/HF energies and the measured ASC shifts is that the latter have negligible *B*-dispersion and an order of magnitude larger level splitting over the entire 0–10 T magnetic field range. This implies a reduction of *ω*_c_, as can be revealed from the FD fit, shown in Fig. 3c. The FD fit gives a general matching with experimental shifts and splittings of *e*_0_-*e*_5_ peaks for *B*_bi_ ~ 6 T and a three-fold reduction of *ω*_c_. The total internal field *B* = *B*_bi_ + *B*_e_ thus has the range *B* = 6–16 T, which includes *ν* = ½ and ¼.

The matching, however, does not account for the appearance of the additional peak *e*_6_ and the ~ 0.2 meV variations of the experimental *B*-dispersion, taking place near *B* ~ 7, 10 and 14 T. These features correspond to the peculiarities of *E*_Coul,rel_(*B*) in Fig. 3d at *ν* = ½ , 1/3 , 2/7 and ¼ and, thus, can be interpreted as the experimental signatures of these states. The appearance of the *e*_6_ peak could indicate crossing of single-*e* *l*_z_ levels near steps in *L*_z_^MN^.

Reduction of *ω*_c_ can be interpreted as fractional charge and manifests a self-generation of the magnetic-quanta-flux vortexes by single *e*s forming *e*^*ν*^s ^35^. The vortex self-generation arises because of the reduction of *E*_tot_ parts *E*_Coul,c.m_ + *E*_cycl_ caused by the decrease in charge and the dissipationless (superconducting) motion of *e*s occupying quantum confined states for *r*_s_ > 2. Such a reduction of *E*_tot_ for *e*^*ν*^s compared to *e*s is shown in Fig. 3e. It reveals a ~ 10 meV minimum at *ν* *=* 5/3 and a gradual decrease from 100 meV for *ν*~ 5/7 to 50 meV for *ν* ~ 1/4, which corresponds to increase of the energy drop from 10 to 80 meV.

### Size dependence of molecular structure

Since the vortexes have fixed radius ~ *a*_B_* ^35^, the resulting charge, i.e., *ν*, *B*_bi_, and AM configuration, are determined mostly by *r*_s_. The size of the *e*^*ν*^ area *d*_*ν*_ increases by a fraction ~ 0.1 per vortex, as can be found from simple geometrical drawing (see the corresponding cartoons for *ν* = ½, 1/3 and ¼ *e*^*ν*^ areas in Fig. 3e). The *d*_*ν*_ value of ~ 40 nm comes from the calculations for *ν* = 1/4 is in agreement with the measured *d*_EA_ in the dot D4e. Thus, this dot can be considered as 6*e*^1/4^ and 5*e*^1/4^ AM in PS and IS, respectively. For the dot D1e a pairing of EAs in IS indicates 3*e*^2/7^ AM, which corresponds to *B*_bi_ ~ 11 T. For *B*_bi_ ~ 6 T of the dot D1d the PS state is 2*e*^3/5^ AM.

In Fig. 3f we present expected schematic arrangements of *e*^*ν*^s in these three AMs obtained by placing vortexes onto the maxima of their *e* distribution. The *e*^3/5^ and *e*^2/7^ are realizations of the complex *e*^*ν*^s, in which *ν* *=* *n*/*p* and *n* > 1, consisting of *n-e*s fused by a “coupling” vortex (see below). The figure accounts for different *e* distribution size, i.e., *r*_s_, and demonstrates matching of *ν* and *r*_s_. This justifies our analysis, which, being extended to the rest of the dots, results in the values *ν* and *B*_bi_ and the *e*^*ν*^-AM configurations in the PS given in Table 1. The analysis shows factor-of-two increase of *B*_bi_ for a 20% increase of *r*_s_ in agreement with the experiment (see Table 1).

### Magneto-electrons and topological quantum computations

**Figure 4:**
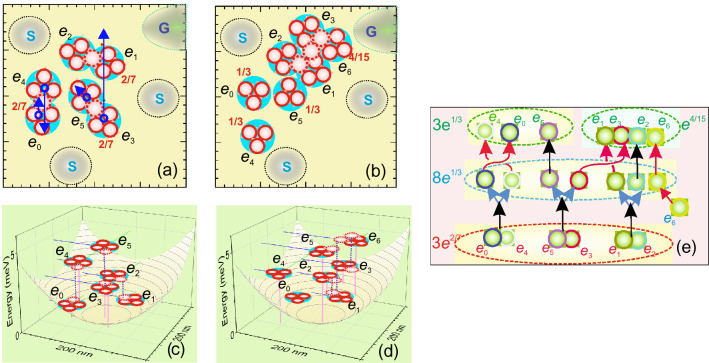
Arrangement of *e*^*ν*^s (large/small circles are electrons/vortexes, dashed circles are coupling vortexes, numbers are fractional charge) in 6*e*-IS (**a)** and 7*e*-PS **(b)** of the dot D1e, respectively and adjacent nano-circuit elements (charge sensors S and gate electrode G). Arrows are displacement vectors of corresponding *e*^*ν*^s, respectively. Energy-space diagrams overlaid on illustrative parabolic confinement potential, respectively, **(c, d)** . Dashed vertical lines outline *e*^*ν*^ coupling. Diagram of world lines of *B*_7_-group describing a PL process of D1e QD **(e)**.

While the AM configurations observed and analyzed in 6*e* states show symmetric, homogenous molecular structures compatible with distorted five- and sixfold circular symmetry and with the calculated electron density distributions, for 7*e* state they are not. The observed anomalous 1D composite configuration for the 7*e* state in the dot D1e (see Fig. 2d) can be assigned to a decomposition of a symmetrical 3*e*^2/7^ AM of a 6*e* state after an extra *e* is added. In this case, the additional *e*^2/7^ needed for symmetrical arrangement, cannot be generated, since it requires pair of *e*s. Thus, we can suppose that the additional *e* creates *e*^1/3^, which results to a transition from 21 to 24 vortexes state. The corresponding configuration could then consists of a 3*e*^1/3^ AM and a single *e*^4/15^. The corresponding arrangements of the *e*^*ν*^s are presented in Figs. 4a,b. In the figures the coupling vortexes are shown by dashed circle.

Comparison of the arrangements and particle displacements in the scheme of Fig. 4a,b reveals transformations and interchange of *e*^*ν*^s, which correspond to elementary topological quantum computing operations (TQCOs). These involve first, two unfusions of (*e*_0_–*e*_4_) and (*e*_3_–*e*_5_) *e*^2/7^ anyon pairs resulting in four *e*^1/3^ single anyons; second, two braids of (*e*_0_,*e*_4_) and (*e*_1_,*e*_3_) pairs; and, third, two fusions, which are a fusion of a *e*^1/3^ (*e*_3_,*e*_6)_ pair forming a *e*^2/7^ anyon and a fusion of a *e*^2/7^ (*e*_1_–*e*_2_) and a (*e*_3_–*e*_6_) pair forming *e*^4/15^ anyon.

The above configuration transformation involves spatially separated *e*^*ν*^-anyons localized on the specific levels of QD confinement potential and a full description of such a transformation should specify a localization energy, i.e., include the energy coordinate. Along these lines we add an energy coordinate to *x–y* plots and present the energy/space diagrams in Fig. 4c,d overlaid on the confinement potential. The diagrams outline a few meV difference in the confinement energy of coupled *e*^*ν*^s.

They show that the coupling vortex should be presented by a pair of half-flux vortexes synchronously generated in gapped *e*^*ν*^-states. These vortexes should have twice size (not shown in the figure) and thus embrace one of the neighboring vortexes. While such a representation of complex *e*^*ν*^s seems unusual in the framework of conventional theories of FQHE states, assuming degenerate states and zeros of many-electron wave-functions for the vortex description^34^, it is supported by our data and by the experimental observations of 2/5 anyons by Aharonov-Bhom interferometry in quantum Hall bars^48^, giving independent evidence of their existence.

TQCOs resulting from the PL process in the dot D1e, presented in the Fig. 4a,b, are described by a *B*_7_ braid group diagram, i.e., time-position world lines, shown in Fig. 4e. The braid of *e*_4_–*e*_0_ and *e*_4_–*e*_0_ *e*^1/3^-anyons should result in a $\Pi$/3 phase change ^23^, which according to the diagram, is encoded in the unfusion of the corresponding *e*^2/7^ anyon and accompanied by a local charge redistribution. This allows one to use local charge sensing to control TQCOs, which can be provided with high sensitivity by a single-*e* transistor (SET)^49^. Since control of phase is a key step in TQCOs and a technology and methodology of SET fabrication and measurements are well developed^50^, using SETs opens up a new possibility for physical realization of TQGs.

The design of the TQG based on InP/GaInP2 *e*^*ν*^-AMs will include few SETs and nano-electrode gates having sizes ~ 50 nm adjacent to individual QDs formed by semiconductor manufacturing technology using *e*-beam lithography ^51^. The arrays of QDs (see Fig. 2e)] can be used to realize circuits. Since we demonstrated that *e*^*ν*^-AM structure is sensitive to small variation of *r*_s_, using nano-gates changing local potentials near QD gives possibility of the fine tuning of the initial state.

In Fig. 4a,b we draw three SETs and a gate near D1e QD, which can be used to realize a prototype of the device for electrical testing of TQCOs shown in Fig. 4e. In the device RF SETs acting simultaneously and utilizing a single RF line by means of carrier multiplexing ^52^ will be used to detect "charge triangulation". Each of the devices is biased at the slope of SET transfer characteristic and employs charge cancellation technique^53^ to minimize effects of direct capacitive coupling to the pulsing gate. In this way signals obtained from SETs (*U*s^*i*^) under variation of *r*_s_ or injection/removing of the electrons by the gate voltage (*U*_G_), i.e., *U*s^*i*^(*U*_G_)-functions, will be used for designing of topological quantum computing processing.

Finally, we should point out, that the QDs considered do not directly involve NA anyons and MZMs, as suggested in initial TQC proposals. However, our magneto-PL measurements and the preliminary analysis of *N* ~ 8 InP/GaInP_2_ dots having *r*_s_ ~ 1.5 (similar to D04 in the insert in Fig. 1a), which will be published elsewhere, reveal *B*_bi_ ~ 2 T close to *ν* ~ 5/2. This can indicate *e*^1/4^ anyon supporting MZMs, similar to that discussed for corresponding SLL FQHE state. The possibility of forming of the corresponding state in an appropriate QD naturally follows from *e*^*ν*^-*E*_tot_ of in Fig. 3e for *ν*> 1. Moreover, for such dot types we also observed a quasi 1D shape of EA^36^, similar to that in Fig. 2b, which can be a signature of TQCOs. We already demonstrated Coulomb blockade control of *N* in these dots in the range 8–18 having Δ*U*_G_ ~ 0.3 meV per electron and the possible formation of a spin-polarized state for *N* > 15 ^54^^.^

## Conclusion

We have introduced a novel system, which can be used for the realization of TQG. These are *e*^*ν*^-anyon molecules (*e*^*ν*^-AM) naturally formed in self-organized many electron InP/GaInP_2_ QD structures. We demonstrated their promising TQC prospects using high-spatial-resolution magneto-PL measurements of five-to-seven electron QDs having *r*_s_ ~ 2. Our data, together with the quantum mechanical calculations of their electronic structure in a magnetic field and analysis demonstrate a self-formation of *e*^*ν*^ AMs, having *ν* ~ 3/5–1/4, corresponding to a built-in magnetic field of 6–12 T. Our measurements of PL spectra and the mapping of the intensity of individual PL lines gave a direct imaging of molecular configurations and allow the observation of a transformation of a *e*^*ν*^ arrangement in a *e*^2/7^-AM under photo-excitation involving fusion and braiding of the anyons. The observed transformation reveals a significant redistribution of the fractional charge within the dot, which suggests the use of single electron transistor charge sensing to control TQC operations. Our investigations show that InP/GaInP_2_ AM QDs having intrinsic anyon localization at zero external magnetic field combined with charge sensing control of anyon’s states open up novel directions for the realization of TQC.

## Methods

### Magneto-PL measurements and data processing

Spatially-resolved magneto-PL spectra were measured using NSOM operating at 10 K and magnetic fields of up to 10 T and using optical fiber probes having an aperture size of 50–300 nm in a collection-illumination mode. The spectra were excited by the 514.5 nm Ar-laser line and measured using a CCD (multi-channel) detector together with a 280 mm focal length monochromator. The excitation power measured before the fiber coupler was ~ 5 µW, which provided a power density of ~ 0.5 W/cm^2^. The spectral resolution of the system is 0.2–0.4 meV.

For the Lorenz contour deconvolution of PL spectra, we used a multi-peak fitting procedure from Origin 8.0 graphic software.

The spatially-resolved PL intensity at the selected wavelengths (image) was generated using the spectra taken in a square grid having a mesh of 50 nm. We plotted the experimental data using a contour plot option of Origin 8.0 and the division of the intensity data into 20 levels. The size of the emission area was estimated as a size of a NSOM image at the emission intensity level of 0.9, i.e. two upper contour plot levels. NSOM image of ASC are related to PS Stokes components images correspond to a difference of electron positions in the IS and PS^36^.

### Configuration interaction and Hartree–Fock many-body calculations

The states of $N$ confined electrons (*N* ~ 6 and *r*_s_ ~ 2.5) in a strong perpendicular magnetic field ${B}_{z}=B$ are modeled theoretically by configuration interaction (CI), using a method described in more detail in Ref. ^36^. We assume a quasi-2D effective-mass approximation with Hamiltonian (in atomic units, $4\pi {\epsilon }_{0}={m}_{e}=\hslash =\left|e\right|=1$)

$$H=\sum_{i=1}^{N}\left\{\frac{1}{2{m}^{*}}{\left[{\mathbf{p}}_{i}-e\mathbf{A}\left({\mathbf{r}}_{i}\right)\right]}^{2}+{V}_{\mathrm{ext}}\left({\mathbf{r}}_{i}\right)+{g}^{*}{{\mu }_{B}B}_{z}{S}_{zi}\right\}+\sum_{i>j}^{N}\frac{{e}^{2}}{\kappa \left|{\mathbf{r}}_{i}-{\mathbf{r}}_{j}\right|} ,$$

where the magnetic vector potential is given by $\mathbf{A}\left(x,y\right)=\left({B}_{z}/2\right)\left(-y,x,0\right)$, and the effective mass ${m}^{*}=0.077$ and dielectric constant $\kappa =12.61$ are taken to be the values for bulk InP. The effective 2D confining potential ${V}_{\mathrm{ext}}\left(\mathbf{r}\right)$ in the $x$-$y$ plane is assumed to be harmonic near the center, with frequency parameter $\hslash {\omega }_{0}$, and to develop a “hard wall” near the physical boundary of the dot on the substrate. The confining potential also has a slight angular deformation, indicated by the potential contours in Fig. 3a, which was chosen to be typical of the dots synthesized experimentally. The spin Zeeman term ${g}^{*}{{\mu }_{B}B}_{z}{S}_{zi}$ is usually not included explicitly. The first step in the CI calculation is to calculate a single-particle basis set $|i\rangle$ using spin-polarized Hartree–Fock (HF),

$$\left[\frac{1}{2{m}^{*}}{\left({\varvec{p}}-e\mathbf{A}\right)}^{2}+{V}_{\mathrm{ext}}+{V}_{\mathrm{HF}}\right]\left|i\right.\rangle ={\epsilon }_{i}\left|i\right.\rangle .$$

The lowest $N$ HF orbitals $|a\rangle$ (where $a=1, \dots , N$) are occupied, with one electron per orbital assuming complete spin polarization. The occupied orbitals contribute to the self-consistent HF potential ${V}_{\mathrm{HF}}$, which is defined as

$$\left.\langle j\right|{V}_{\mathrm{HF}}\left|i\right.\rangle =\frac{{e}^{2}}{\kappa }\sum_{a}^{\mathrm{occ}}\left(\langle ja\left|{r}_{12}^{-1}\right|ia\rangle -\langle ja\left|{r}_{12}^{-1}\right|ai\rangle \right) ,$$

where $|i\rangle$ and $|j\rangle$ are general states (occupied or unoccupied). The eigenvalues ${\epsilon }_{a}$ of the occupied orbitals, at least for zero to moderate ${B}_{z}$, may be expected to give approximations to the removal energies of electrons from the system, according to Koopman’s theorem from atomic and molecular physics^54^. In the calculated electron density distributions the dot size (*D*_c_) was estimated as the size of the area containing 96% of electron density.
